# Supplementary material for: What Motivates Patients with COPD to Be Physically Active? A Cross-Sectional Study
Source: J Clin Med. 2021 Nov 29;10(23):5631. doi: 10.3390/jcm10235631 (PMC8658241; doi:10.3390/jcm10235631)
Supplement: Supplementary file 1 [file jcm-10-05631-s001.zip › jcm-1463903-supplementary.pdf]

Supplementary Material

1 Cronbach's alpha for the Exercise Inventory Motivation 2 (EMI-2) 2
2 Sociodemographic and clinical variables 3
2.1 Valid data 3
3 Motivational Factors and dimensions of the Exercise Motivation Inventory (EMI-2) among PR participation groups 4
3.1 Valid data 4
4 Motivational dimensions and factors of the Exercise Motivation Inventory (EMI-2) among Sedentary and Active lifestyle groups 5
4.1 Valid data 5
5 Motivational Factors and dimensions of the Exercise Motivation Inventory (EMI-2) among GOLD 1-4 groups 6
5.1 Valid data 6
6 Motivational Factors and dimensions of the Exercise Motivation Inventory (EMI-2) among GOLD ABCD groups 7
6.1 Valid data 7
7 Motivational Factors and dimensions of the Exercise Motivation Inventory (EMI-2) among mMRC groups 8
7.1 Valid data 8
8 Motivational Factors and dimensions of the Exercise Motivation Inventory (EMI-2) among CAT groups 9
8.1 Valid data 9
9 Motivational Factors and dimensions of the Exercise Motivation Inventory (EMI-2) among CIS20-SF groups 10
9.1 Valid data 10
10 Motivational Factors and dimensions of the Exercise Motivation Inventory (EMI-2) among 6MWT groups 11
10.1 Valid data 11
11 Motivational Factors and dimensions of the Exercise Motivation Inventory (EMI-2) among comorbidities groups 12
11.1 Valid data 12

1 Cronbach's alpha for the Exercise Inventory Motivation 2 (EMI-2)

Table S1. Cronbach's alpha for the Exercise Inventory Motivation 2 (EMI-2).

**Motivational Factors and dimensions**	**Cronbach's α**
**Psychological Motives**	**0.905**
Stress Management	0.804
Revitalisation	0.751
Enjoyment	0.890
Challenge	0.829
**Interpersonal Motives**	**0.868**
Social Recognition	0.867
Affiliation	0.841
Competition	0.879
**Health Motives**	**0.861**
Health Pressure	0.559
Ill-Health Avoidance	0.781
Positive Health	0.860
**Body Related Motives**	**0.721**
Weight Management	0.787
Appearance	0.898
**Fitness Motives**	0.880
Strength/Endurance	0.814
Nimbleness	0.843

2 Sociodemographic and clinical variables

2.1 Valid data

Table S2. Sample sizes for sociodemographic and clinical variables, in total and Pulmonary Rehabilitation (PR) participation groups.

	Total sample	Never PR	Previous PR	Current PR
**Age**	92	28	47	17
**Sex**	92	28	47	17
**Time since last PR**	-	-	44	-
**No. sessions attended**	-	-	-	12
**Education**	92	28	47	17
**Smoking status**	92	28	47	17
**BMI (kg/m2)**	92	28	47	17
**FEV_1 (% predicted)**	91	27	47	17
**FVC (% predicted)**	91	27	47	17
**FEV_1/FVC**	91	27	47	17
**GOLD 1–4**	91	27	47	17
**GOLD ABCD**	88	27	46	15
**Comorbidities**	92	28	47	17
**Hypertension**	91	28	46	17
**Dyslipidemia**	92	28	47	17
**Anxiety**	92	28	47	17
**Diabetes Mellitus**	92	28	47	17
**Dyspnea (mMRC)**	91	28	46	17
**CAT**	91	28	47	16
**CIS20-SF**	88	26	45	17
**6MWD**	90	28	45	17
**6MWD % predicted**	90	28	45	17
**Physical activity (MVPA, steps/day)**	90	27	46	17
**Physical activity (PA) category**	90	27	46	17

Abbreviations: BMI, Body Mass Index; CAT, COPD Assessment Test; CIS20-SF, Checklist of Individual Strength – Short Form; FEV_1, Forced Expiratory Volume in First Second; FVC, Forced Vital Capacity; GOLD, Global Initiative for Obstructive Lung Disease; mMRC, Modified Medical Research Council; MVPA, moderate-to-vigorous physical activity; PR, Pulmonary Rehabilitation; 6MWD, Six-Minute Walking Distance.

3 Motivational Factors and dimensions of the Exercise Motivation Inventory (EMI-2) among PR participation groups

Table S3. Motivational Factors (in bold) and dimensions of the EMI-2 in PR participation groups.

Motivational Factors and dimensions	Total sample	Never PR	Previous PR	Current PR	p-value
**Psychological Motives**	2.9 (1.3)	2.6 (1.5)	3.2 (1.2)	2.8 (1.2)	0.137 [a]
Stress Management	2.7 (1.5)	2.6 (1.5)	2.8 (1.5)	2.4 (1.4)	0.489 [b]
Revitalisation	3.6 (1.3)	3.3 (1.5)	3.8 (1.1)	3.5 (1.1)	0.187 [b]
Enjoyment	2.9 (1.5)	2.4 (1.7)	3.3 (1.3)	2.8 (1.3)	0.047* [b,c]
Challenge	2.6 (1.5)	2.4 (1.7)	2.8 (1.4)	2.7 (1.4)	0.565 [a]
**Interpersonal Motives**	1.7 (1.4)	1.5 (1.3)	2.0 (1.5)	1.5 (1.1)	0.372 [b]
Social Recognition	1.4 (1.5)	1.1 (1.5)	1.6 (1.6)	1.2 (1.3)	0.513 [b]
Affiliation	2.1 (1.5)	2.0 (1.7)	2.3 (1.5)	1.7 (1.3)	0.319 [b]
Competition	1.7 (1.6)	1.3 (1.6)	2.0 (1.8)	1.5 (1.1)	0.194 [b]
**Health Motives**	3.8 (1.1)	3.7 (1.1)	3.8 (1.1)	3.8 (1.1)	0.941 [b]
Health Pressure	3.4 (1.3)	3.3 (1.6)	3.4 (1.2)	3.6 (1.0)	0.897 [b]
Ill-Health Avoidance	3.9 (1.2)	3.9 (1.1)	4.0 (1.2)	3.8 (1.3)	0.648 [b]
Positive Health	4.0 (1.2)	4.0 (1.2)	4.1 (1.2)	4.0 (1.4)	0.920 [b]
**Body Related Motives**	2.0 (1.4)	1.9 (1.4)	2.1 (1.5)	2.0 (1.2)	0.710 [a]
Weight Management	2.4 (1.5)	2.5 (1.6)	2.4 (1.5)	2.4 (1.4)	0.973 [b]
Appearance	1.7 (1.7)	1.3 (1.5)	1.9 (1.8)	1.6 (1.4)	0.425 [b]
**Fitness Motives**	3.4 (1.3)	3.3 (1.4)	3.6 (1.2)	3.3 (1.3)	0.607 [b]
Strength/Endurance	3.4 (1.3)	3.2 (1.4)	3.6 (1.3)	3.1 (1.4)	0.324 [b]
Nimbleness	3.5 (1.4)	3.4 (1.5)	3.5 (1.4)	3.5 (1.3)	0.845 [b]

Data presented as mean (standard deviation). Abbreviations: PR – Pulmonary Rehabilitation. [a] ANOVA test. [b] Kruskall-Wallis test. [c] Post-hoc pairwise comparisons with Bonferroni correction: 'Never PR' and 'Previous PR' (p=0.055). * Difference between groups significant at $p < 0.05$.

3.1 Valid data

Table S4. Sample size per Motivational Factor and Dimension of the EMI-2 in total sample and PR groups.

Motivational Factors and dimensions	Total sample	Never PR	Previous PR	Current PR
**Psychological Motives**	84	26	41	17
Stress Management	87	27	43	17
Revitalisation	89	27	45	17
Enjoyment	90	28	45	17
Challenge	89	28	44	17
**Interpersonal Motives**	88	28	45	15
Social Recognition	89	28	46	15
Affiliation	92	28	47	17
Competition	91	28	46	17
**Health Motives**	89	28	44	17
Health Pressure	91	28	46	17
Ill-Health Avoidance	92	28	47	17
Positive Health	89	28	44	17
**Body Related Motives**	90	27	46	17
Weight Management	91	28	46	17
Appearance	91	27	47	17
**Fitness Motives**	91	28	47	16
Strength/Endurance	91	28	47	16
Nimbleness	92	18	47	17

4 Motivational dimensions and factors of the Exercise Motivation Inventory (EMI-2) among Sedentary and Active lifestyle groups

Table S5. Motivational dimensions (in bold) and factors of the EMI-2 in the Sedentary and Active lifestyle groups.

Motivational dimensions and factors	Sedentary Lifestyle (<5000 steps/day)	Active lifestyle (≥5000 steps/day)	p-value
**Psychological Motives**	2.8 (1.4)	3.0 (1.2)	0.449 [a]
Stress Management	2.6 (1.6)	2.8 (1.3)	0.609 [a]
Revitalization	3.4 (1.3)	3.8 (1.3)	0.190 [a]
Enjoyment	2.8 (1.6)	3.1 (1.5)	0.343 [a]
Challenge	2.5 (1.6)	2.8 (1.5)	0.312 [b]
**Interpersonal Motives**	1.7 (1.3)	1.7 (1.5)	0.658 [a]
Social Recognition	1.4 (1.5)	1.4 (1.6)	0.910 [a]
Affiliation	2.1 (1.6)	2.1 (1.5)	0.909 [a]
Competition	1.7 (1.6)	1.7 (1.8)	0.683 [a]
**Health Motives**	3.8 (1.2)	3.8 (1.1)	0.834 [a]
Health Pressure	3.4 (1.3)	3.4 (1.4)	0.970 [a]
Health Avoid	3.9 (1.3)	4.0 (1.1)	0.898 [a]
Positive Health	3.9 (1.3)	4.2 (1.1)	0.561 [a]
**Body Related Motives**	1.9 (1.4)	2.1 (1.4)	0.554 [b]
Weight Management	2.3 (1.5)	2.5 (1.5)	0.561 [a]
Appearance	1.7 (1.8)	1.7 (1.6)	0.576 [a]
**Fitness Motives**	3.4 (1.4)	3.5 (1.2)	0.908 [a]
Strength/Endurance	3.4 (1.4)	3.4 (1.3)	0.734 [a]
Nimbleness	3.4 (1.5)	3.6 (1.3)	0.675 [a]

Data presented as mean (standard deviation). [a] Mann-Whitney test. [b] Independent samples t-test.

4.1 Valid data

Table S6. Sample size per Motivational Factor and Dimension of the EMI-2 in the Sedentary and Active lifestyle groups.

Motivational Factors and dimensions	Total	Sedentary (<5000 steps/day)	Active (≥5000 steps/day)
**Psychological Motives**	82	43	39
Stress Management	85	44	41
Revitalisation	87	47	40
Enjoyment	88	47	41
Challenge	87	47	40
**Interpersonal Motives**	86	47	39
Social Recognition	87	47	40
Affiliation	90	49	41
Competition	89	49	40
**Health Motives**	87	47	40
Health Pressure	89	48	41
Ill-Health Avoidance	90	49	41
Positive Health	87	47	40
**Body Related Motives**	88	47	41
Weight Management	89	48	41
Appearance	89	48	41
**Fitness Motives**	89	49	40
Strength/Endurance	89	49	40
Nimbleness	90	49	41

5 Motivational Factors and dimensions of the Exercise Motivation Inventory (EMI-2) among GOLD 1-4 groups

Table S7. Motivational Factors (in bold) and dimensions of the EMI-2 in the GOLD 1-4 groups.

	GOLD				
**Motivational Factors and dimensions**	**1**	**2**	**3**	**4**	**p-value** [a]
**Psychological Motives**	3.2 (1.8)	2.8 (1.2)	3.0 (1.3)	2.7 (1.3)	0.665
Stress Management	3.6 (1.4)	2.7 (1.5)	2.7 (1.4)	2.5 (1.6)	0.692
Revitalisation	3.0 (1.6)	3.6 (1.2)	3.7 (1.3)	3.3 (1.3)	0.334
Enjoyment	2.9 (2.1)	2.9 (1.5)	3.0 (1.6)	2.7 (1.4)	0.861
Challenge	3.3 (2.1)	2.4 (1.5)	2.8 (1.5)	2.6 (1.4)	0.535
**Interpersonal Motives**	2.9 (2.2)	1.6 (1.1)	1.7 (1.5)	1.8 (1.6)	0.706
Social Recognition	2.3 (2.3)	1.2 (1.3)	1.4 (1.6)	1.6 (1.7)	0.722
Affiliation	3.2 (2.3)	2.0 (1.4)	2.1 (1.6)	1.9 (1.5)	0.629
Competition	3.1 (2.3)	1.6 (1.4)	1.6 (1.8)	1.7 (1.5)	0.498
**Health Motives**	3.8 (1.2)	3.9 (0.9)	3.8 (1.2)	3.5 (1.2)	0.761
Health Pressure	3.7 (1.4)	3.3 (1.3)	3.6 (1.3)	3.2 (1.4)	0.553
Ill-Health Avoidance	3.9 (1.2)	4.1 (1.0)	3.9 (1.4)	3.6 (1.3)	0.649
Positive Health	3.8 (1.3)	4.2 (1.0)	4.0 (1.4)	3.8 (1.3)	0.903
**Body Related Motives**	2.7 (2.1)	2.2 (1.2)	1.8 (1.5)	2.0 (1.3)	0.692
Weight Management	2.6 (2.1)	2.9 (1.4)	2.0 (1.5)	2.3 (1.3)	0.156
Appearance	2.8 (2.2)	1.6 (1.5)	1.6 (1.7)	1.7 (1.8)	0.725
**Fitness Motives**	3.1 (1.7)	3.5 (1.1)	3.5 (1.5)	3.3 (1.3)	0.863
Strength/Endurance	3.1 (1.7)	3.5 (1.1)	3.4 (1.5)	3.3 (1.4)	0.947
Nimbleness	3.2 (2.0)	3.4 (1.2)	3.6 (1.5)	3.4 (1.4)	0.584

Data presented as mean (standard deviation). [a] Kruskall-Wallis test.

5.1 Valid data

Table S8. Sample size per Motivational Factor and Dimension of the EMI-2 in the GOLD 1-4 groups.

	Total	GOLD			
**Motivational Factors and dimensions**		**1**	**2**	**3**	**4**
**Psychological Motives**	83	4	30	33	16
Stress Management	86	4	31	35	16
Revitalisation	88	4	33	35	16
Enjoyment	89	4	33	36	16
Challenge	88	4	33	35	16
**Interpersonal Motives**	87	4	35	35	13
Social Recognition	88	4	35	35	14
Affiliation	91	4	35	36	16
Competition	90	4	35	36	15
**Health Motives**	88	4	33	35	16
Health Pressure	90	4	35	35	16
Ill-Health Avoidance	91	4	35	36	16
Positive Health	88	4	33	35	16
**Body Related Motives**	89	4	33	36	16
Weight Management	90	4	34	36	16
Appearance	90	4	34	36	16
**Fitness Motives**	90	4	35	36	15
Strength/Endurance	90	4	35	36	15
Nimbleness	91	4	35	36	16

6 Motivational Factors and dimensions of the Exercise Motivation Inventory (EMI-2) among GOLD ABCD groups

Table S9. Motivational Factors (in bold) and dimensions of the EMI-2 in the GOLD ABCD groups.

	GOLD				
**Motivational Factors and dimensions**	**A**	**B**	**C**	**D**	**p-value**
**Psychological Motives**	3.1 (1.0)	2.6 (1.7)	3.1 (1.2)	2.8 (1.5)	0.650 [a]
Stress Management	2.9 (1.3)	2.3 (1.6)	2.7 (1.4)	2.8 (1.7)	0.131 [b]
Revitalisation	4.0 (1.0)	2.9 (1.6)	3.6 (1.1)	3.5 (1.3)	0.530 [b]
Enjoyment	3.2 (1.3)	2.5 (1.9)	3.1 (1.5)	2.7 (1.6)	0.540 [b]
Challenge	2.6 (1.4)	2.4 (1.7)	3.1 (1.6)	2.8 (1.6)	0.816 [b]
**Interpersonal Motives**	1.8 (1.3)	1.7 (1.7)	1.7 (1.3)	1.9 (1.7)	0.965 [b]
Social Recognition	1.3 (1.4)	1.7 (1.7)	1.3 (1.4)	1.7 (1.9)	0.889 [b]
Affiliation	2.3 (1.5)	1.9 (1.7)	1.9 (1.4)	2.3 (1.7)	0.766 [b]
Competition	1.8 (1.5)	1.5 (1.7)	1.9 (1.9)	1.8 (1.8)	0.930 [a]
**Health Motives**	4.0 (0.9)	3.5 (1.4)	3.8 (0.9)	3.7 (1.2)	0.579 [b]
Health Pressure	3.6 (1.2)	3.2 (1.6)	3.5 (1.4)	3.4 (1.4)	0.695 [a]
Ill-Health Avoidance	4.2 (1.0)	3.7 (1.4)	3.8 (1.1)	3.8 (1.4)	0.405 [b]
Positive Health	4.3 (1.0)	3.6 (1.6)	4.2 (1.0)	3.9 (1.4)	0.402 [b]
**Body Related Motives**	2.2 (1.4)	1.8 (1.5)	1.9 (1.0)	2.0 (1.8)	0.843 [b]
Weight Management	2.7 (1.5)	2.0 (1.5)	2.2 (1.1)	2.3 (1.8)	0.368 [b]
Appearance	1.7 (1.6)	1.8 (1.9)	1.7 (1.3)	1.8 (2.0)	0.997 [b]
**Fitness Motives**	3.5 (1.1)	3.0 (1.7)	3.9 (0.8)	3.4 (1.6)	0.682 [b]
Strength/Endurance	3.5 (1.1)	3.0 (1.7)	4.0 (1.0)	3.3 (1.7)	0.384 [b]
Nimbleness	3.5 (1.3)	3.1 (1.8)	3.8 (1.0)	3.6 (1.6)	0.678 [b]

Data presented as mean (standard deviation). [a] ANOVA Test; [b] Kruskall-Wallis test.

6.1 Valid data

Table S10. Sample size per Motivational Factor and Dimension of the EMI-2 in the GOLD ABCD groups.

	Total	GOLD			
**Motivational Factors and dimensions**		**A**	**B**	**C**	**D**
**Psychological Motives**	81	39	13	14	15
Stress Management	84	40	14	14	16
Revitalisation	85	40	15	14	16
Enjoyment	86	40	15	14	17
Challenge	85	39	15	14	17
**Interpersonal Motives**	84	38	15	15	16
Social Recognition	85	39	15	15	16
Affiliation	88	40	16	15	17
Competition	87	39	16	15	17
**Health Motives**	86	39	15	15	17
Health Pressure	87	40	15	15	17
Ill-Health Avoidance	88	40	16	15	17
Positive Health	86	39	15	15	17
**Body Related Motives**	86	40	15	15	16
Weight Management	87	40	15	15	17
Appearance	87	40	16	15	16
**Fitness Motives**	87	39	16	15	17
Strength/Endurance	87	39	16	15	17
Nimbleness	88	40	16	15	17

7 Motivational Factors and dimensions of the Exercise Motivation Inventory (EMI-2) among mMRC groups

Table S11. Motivational Factors (in bold) and dimensions of the EMI-2 in the mMRC groups.

	mMRC		
**Motivational Factors and dimensions**	**mMRC < 2**	**mMRC ≥ 2**	**p-value**
**Psychological Motives**	3.0 (1.1)	2.8 (1.5)	0.539 [a]
Stress Management	2.8 (1.4)	2.6 (1.6)	0.484 [b]
Revitalisation	3.9 (1.0)	3.1 (1.4)	0.017* [a]
Enjoyment	3.1 (1.3)	2.7 (1.7)	0.331 [a]
Challenge	2.6 (1.4)	2.7 (1.6)	0.703 [a]
**Interpersonal Motives**	1.7 (1.3)	1.9 (1.6)	0.798 [a]
Social Recognition	1.2 (1.4)	1.6 (1.7)	0.282 [a]
Affiliation	2.1 (1.5)	2.1 (1.6)	0.814 [a]
Competition	1.6 (1.5)	1.8 (1.8)	0.695 [a]
**Health Motives**	4.0 (0.9)	3.6 (1.3)	0.221 [a]
Health Pressure	3.5 (1.2)	3.3 (1.4)	0.533 [a]
Ill-Health Avoidance	4.1 (1.0)	3.7 (1.4)	0.191 [a]
Positive Health	4.3 (1.0)	3.7 (1.4)	0.049* [a]
**Body Related Motives**	2.1 (1.3)	1.9 (1.5)	0.501 [b]
Weight Management	2.6 (1.5)	2.2 (1.5)	0.168 [a]
Appearance	1.6 (1.6)	1.8 (1.8)	0.908 [a]
**Fitness Motives**	3.6 (1.0)	3.2 (1.5)	0.449 [a]
Strength/Endurance	3.6 (1.1)	3.2 (1.6)	0.359 [a]
Nimbleness	3.6 (1.2)	3.3 (1.6)	0.785 [a]

Data presented as mean (standard deviation). [a] Mann-Whitney test. [b] Independent samples t-test. * Difference between groups significant at $p < 0.05$.

7.1 Valid data

Table S12. Sample size per Motivational Factor and Dimension of the EMI-2 in the mMRC groups.

	Total	mMRC	
**Motivational Factors and dimensions**		**mMRC < 2**	**mMRC ≥ 2**
**Psychological Motives**	84	48	36
Stress Management	87	49	38
Revitalisation	88	49	39
Enjoyment	89	49	40
Challenge	88	48	40
**Interpersonal Motives**	87	47	40
Social Recognition	88	48	40
Affiliation	91	49	42
Competition	90	48	42
**Health Motives**	89	48	41
Health Pressure	90	49	41
Ill-Health Avoidance	91	49	42
Positive Health	89	48	41
**Body Related Motives**	89	49	40
Weight Management	90	49	41
Appearance	90	49	41
**Fitness Motives**	90	48	42
Strength/Endurance	90	48	42
Nimbleness	91	49	42

8 Motivational Factors and dimensions of the Exercise Motivation Inventory (EMI-2) among CAT groups

Table S13. Motivational Factors (in bold) and dimensions of the EMI-2 in the CAT groups.

	CAT		
**Motivational Factors and dimensions**	**CAT < 10**	**CAT ≥ 10**	**p-value**
**Psychological Motives**	3.3 (0.8)	2.8 (1.4)	0.157 [a]
Stress Management	2.9 (1.4)	2.6 (1.5)	0.295 [a]
Revitalisation	4.2 (0.6)	3.3 (1.3)	0.009** [a]
Enjoyment	3.5 (1.0)	2.7 (1.6)	0.048* [a]
Challenge	2.7 (1.4)	2.6 (1.5)	0.699 [b]
**Interpersonal Motives**	1.8 (1.2)	1.7 (1.5)	0.266 [a]
Social Recognition	1.2 (1.4)	1.4 (1.6)	0.683 [a]
Affiliation	2.6 (1.2)	1.9 (1.6)	0.021* [a]
Competition	1.7 (1.7)	1.7 (1.6)	0.808 [a]
**Health Motives**	4.2 (0.7)	3.6 (1.2)	0.061 [a]
Health Pressure	3.7 (1.2)	3.3 (1.3)	0.209 [a]
Ill-Health Avoidance	4.4 (0.8)	3.8 (1.3)	0.034* [a]
Positive Health	4.6 (0.7)	3.8 (1.3)	0.016* [a]
**Body Related Motives**	2.2 (1.4)	2.0 (1.4)	0.542 [b]
Weight Management	2.6 (1.5)	2.4 (1.5)	0.449 [a]
Appearance	1.8 (1.7)	1.6 (1.6)	0.561 [a]
**Fitness Motives**	3.8 (0.7)	3.3 (1.4)	0.124 [a]
Strength/Endurance	3.8 (0.7)	3.2 (1.5)	0.250 [a]
Nimbleness	3.9 (1.0)	3.3 (1.5)	0.093 [a]

Data presented as mean (standard deviation). Abbreviations: CAT – COPD Assessment Test. [a] Mann-Whitney test. [b] Independent samples t-test. * Difference between groups significant at $p < 0.05$. ** Difference between groups significant at $p \leq 0.01$.

8.1 Valid data

Table S14. Sample size per Motivational Factor and Dimension of the EMI-2 in the CAT groups.

	Total	CAT	
**Motivational Factors and dimensions**		**CAT < 10**	**CAT ≥ 10**
**Psychological Motives**	83	22	61
Stress Management	86	22	64
Revitalisation	88	22	66
Enjoyment	89	22	67
Challenge	88	23	65
**Interpersonal Motives**	87	23	64
Social Recognition	88	23	65
Affiliation	91	23	68
Competition	90	23	67
**Health Motives**	88	23	65
Health Pressure	90	23	67
Ill-Health Avoidance	91	23	68
Positive Health	88	23	65
**Body Related Motives**	89	22	67
Weight Management	90	22	68
Appearance	90	23	67
**Fitness Motives**	90	23	67
Strength/Endurance	90	23	67
Nimbleness	91	23	68

9 Motivational Factors and dimensions of the Exercise Motivation Inventory (EMI-2) among CIS20-SF groups

Table S15. Motivational Factors (in bold) and dimensions of the EMI-2 in the CIS20-SF groups.

	CIS20-SF		
**Motivational Factors and dimensions**	**Normal Fatigue (CIS20-SF <27)**	**Abnormal Fatigue (CIS20-SF ≥27)**	**p-value**
**Psychological Motives**	3.4 (0.9)	2.8 (1.4)	0.089 [a]
Stress Management	2.7 (1.4)	2.7 (1.5)	0.948 [a]
Revitalisation	4.3 (0.8)	3.3 (1.3)	0.001** [a]
Enjoyment	3.7 (1.1)	2.7 (1.5)	0.005** [b]
Challenge	2.9 (1.4)	2.5 (1.5)	0.240 [b]
**Interpersonal Motives**	1.8 (1.3)	1.7 (1.5)	0.558 [a]
Social Recognition	1.2 (1.4)	1.5 (1.6)	0.562 [a]
Affiliation	2.6 (1.5)	1.9 (1.5)	0.065 [a]
Competition	1.7 (1.7)	1.8 (1.6)	0.650 [a]
**Health Motives**	4.1 (0.8)	3.7 (1.1)	0.245 [a]
Health Pressure	3.4 (1.3)	3.4 (1.3)	0.974 [a]
Ill-Health Avoidance	4.3 (1.1)	3.8 (1.2)	0.037* [a]
Positive Health	4.6 (0.6)	3.9 (1.3)	0.020* [a]
**Body Related Motives**	2.3 (1.1)	2.0 (1.4)	0.403 [b]
Weight Management	2.8 (1.2)	2.3 (1.5)	0.147 [a]
Appearance	1.8 (1.6)	1.7 (1.7)	0.659 [a]
**Fitness Motives**	3.9 (0.9)	3.3 (1.3)	0.057 [a]
Strength/Endurance	3.9 (0.9)	3.3 (1.4)	0.083 [a]
Nimbleness	4.0 (1.1)	3.3 (1.4)	0.058 [a]

Data presented as mean (standard deviation). Abbreviations: CIS20-SF – Checklist of Individual Strength – Subjective Fatigue. [a] Mann-Whitney test. [b] Independent samples t-test. * Difference between groups significant at $p < 0.05$. ** Difference between groups significant at $p \leq 0.01$.

9.1 Valid data

Table S16. Sample size per Motivational Factor and Dimension of the EMI-2 in the CIS20-SF groups.

	Total	CIS20-SF	
**Motivational Factors and dimensions**		**Normal Fatigue (CIS20-SF <27)**	**Abnormal Fatigue (CIS20-SF ≥27)**
**Psychological Motives**	80	25	55
Stress Management	83	25	58
Revitalisation	85	25	60
Enjoyment	86	25	61
Challenge	85	26	59
**Interpersonal Motives**	84	26	58
Social Recognition	85	26	59
Affiliation	88	26	62
Competition	87	26	61
**Health Motives**	85	26	59
Health Pressure	87	26	61
Ill-Health Avoidance	88	26	62
Positive Health	85	26	59
**Body Related Motives**	86	25	61
Weight Management	87	25	62
Appearance	87	26	61
**Fitness Motives**	87	26	61
Strength/Endurance	87	26	61
Nimbleness	88	26	62

10 Motivational Factors and dimensions of the Exercise Motivation Inventory (EMI-2) among 6MWT groups

Table S17. Motivational Factors (in bold) and dimensions of the EMI-2 in 6MWT groups.

	6MWT		
**Motivational Factors and dimensions**	**Distance < 350m**	**Distance ≥ 350m**	**p-value**
**Psychological Motives**	2.9 (1.2)	2.9 (1.3)	0.869 [a]
Stress Management	2.8 (1.3)	2.6 (1.5)	0.692 [a]
Revitalisation	3.4 (1.2)	3.6 (1.3)	0.340 [a]
Enjoyment	3.0 (1.5)	2.9 (1.5)	0.725 [a]
Challenge	2.5 (1.5)	2.6 (1.5)	0.851 [b]
**Interpersonal Motives**	1.7 (1.3)	1.7 (1.4)	0.697 [a]
Social Recognition	1.5 (1.5)	1.3 (1.5)	0.329 [a]
Affiliation	2.0 (1.4)	2.1 (1.6)	0.974 [a]
Competition	1.6 (1.5)	1.7 (1.7)	0.895 [a]
**Health Motives**	3.7 (1.2)	3.8 (1.1)	0.818 [a]
Health Pressure	3.6 (1.2)	3.3 (1.3)	0.544 [a]
Ill-Health Avoidance	3.8 (1.3)	4.0 (1.2)	0.733 [a]
Positive Health	3.8 (1.3)	4.1 (1.2)	0.330 [a]
**Body Related Motives**	1.8 (1.3)	2.1 (1.4)	0.347 [b]
Weight Management	2.1 (1.4)	2.5 (1.5)	0.239 [a]
Appearance	1.6 (1.7)	1.7 (1.6)	0.710 [a]
**Fitness Motives**	3.5 (1.2)	3.4 (1.3)	0.790 [a]
Strength/Endurance	3.6 (1.2)	3.3 (1.4)	0.492 [a]
Nimbleness	3.4 (1.4)	3.5 (1.4)	0.921 [a]

Data presented as mean (standard deviation). Abbreviations: 6MWT – 6 minute walking test. [a] Mann-Whitney test. [b] Independent samples t-test.

10.1 Valid data

Table S18. Sample size per Motivational Factor and Dimension of the EMI-2 in the 6MWT groups.

	Total	6MWT	
**Motivational Factors and dimensions**		**Distance < 350m**	**Distance ≥ 350m**
**Psychological Motives**	82	21	61
Stress Management	85	21	64
Revitalisation	87	22	65
Enjoyment	88	22	66
Challenge	87	22	65
**Interpersonal Motives**	86	20	66
Social Recognition	87	21	66
Affiliation	90	22	68
Competition	89	21	68
**Health Motives**	87	22	65
Health Pressure	89	22	67
Ill-Health Avoidance	90	22	68
Positive Health	87	22	65
**Body Related Motives**	88	21	67
Weight Management	89	22	67
Appearance	89	21	68
**Fitness Motives**	89	22	67
Strength/Endurance	89	22	67
Nimbleness	90	22	68

11 Motivational Factors and dimensions of the Exercise Motivation Inventory (EMI-2) among comorbidities groups

Table S19. Motivational Factors (in bold) and dimensions of the EMI-2 in the comorbidities groups.

Motivational Factors and dimensions	Comorbidities < 2	Comorbidities ≥ 2	p-value
**Psychological Motives**	2.5 (1.5)	3.0 (1.2)	0.189 [a]
Stress Management	2.2 (1.7)	2.8 (1.4)	0.182 [b]
Revitalisation	3.4 (1.6)	3.6 (1.2)	0.944 [b]
Enjoyment	2.7 (1.7)	3.0 (1.5)	0.482 [a]
Challenge	2.3 (1.6)	2.7 (1.5)	0.358 [a]
**Interpersonal Motives**	1.4 (1.3)	1.8 (1.4)	0.193 [b]
Social Recognition	0.9 (1.2)	1.5 (1.6)	0.141 [b]
Affiliation	2.1 (1.8)	2.1 (1.5)	0.781 [b]
Competition	1.1 (1.4)	1.8 (1.7)	0.075 [b]
**Health Motives**	3.8 (1.2)	3.8 (1.1)	0.958 [b]
Health Pressure	3.4 (1.4)	3.4 (1.3)	0.870 [b]
Ill-Health Avoidance	4.0 (1.3)	3.9 (1.2)	0.554 [b]
Positive Health	4.0 (1.4)	4.0 (1.2)	0.931 [b]
**Body Related Motives**	1.3 (1.2)	2.2 (1.4)	0.013* [a]
Weight Management	1.5 (1.3)	2.6 (1.5)	0.005** [b]
Appearance	1.1 (1.6)	1.8 (1.6)	0.031* [b]
**Fitness Motives**	3.3 (1.5)	3.5 (1.2)	0.767 [b]
Strength/Endurance	3.0 (1.6)	3.5 (1.3)	0.268 [b]
Nimbleness	3.6 (1.6)	3.4 (1.3)	0.336 [b]

Data presented as mean (standard deviation). [a] Mann-Whitney test. [b] Independent samples t-test.

11.1 Valid data

Table S20. Sample size per Motivational Factor and Dimension of the EMI-2 in the comorbidities groups.

Motivational Factors and dimensions	Total	Comorbidities < 2	Comorbidities ≥ 2
**Psychological Motives**	84	16	68
Stress Management	87	17	70
Revitalisation	89	16	73
Enjoyment	90	17	73
Challenge	89	17	72
**Interpersonal Motives**	88	17	71
Social Recognition	89	17	72
Affiliation	92	17	75
Competition	91	17	74
**Health Motives**	89	17	72
Health Pressure	91	17	74
Ill-Health Avoidance	92	17	75
Positive Health	89	17	72
**Body Related Motives**	90	17	73
Weight Management	91	17	74
Appearance	91	17	74
**Fitness Motives**	91	17	74
Strength/Endurance	91	17	74
Nimbleness	92	17	75
